# Mass Spectrometric Studies of Apolipoprotein Proteoforms and Their Role in Lipid Metabolism and Type 2 Diabetes

**DOI:** 10.3390/proteomes5040027

**Published:** 2017-10-15

**Authors:** Dobrin Nedelkov

**Affiliations:** Isoformix Inc., Tempe, AZ 85284, USA; dobrin.nedelkov@isoformix.com; Tel.: +1-480-777-7570

**Keywords:** apolipoprotein, mass spectrometry, proteoform, plasma, biomarker

## Abstract

Apolipoproteins function as structural components of lipoprotein particles, cofactors for enzymes, and ligands for cell-surface receptors. Most of the apoliporoteins exhibit proteoforms, arising from single nucleotide polymorphisms (SNPs) and post-translational modifications such as glycosylation, oxidation, and sequence truncations. Reviewed here are recent studies correlating apolipoproteins proteoforms with the specific clinical measures of lipid metabolism and cardiometabolic risk. Targeted mass spectrometric immunoassays toward apolipoproteins A-I, A-II, and C-III were applied on large cross-sectional and longitudinal clinical cohorts. Several correlations were observed, including greater apolipoprotein A-I and A-II oxidation in patients with diabetes and cardiovascular disease, and a divergent apoC-III proteoforms association with plasma triglycerides, indicating significant differences in the metabolism of the individual apoC-III proteoforms. These are the first studies of their kind, correlating specific proteoforms with clinical measures in order to determine their utility as potential clinical biomarkers for disease diagnosis, risk stratification, and therapy decisions. Such studies provide the impetus for the further development and clinical translation of MS-based protein tests.

## 1. Introduction

Protein biomarkers are critical for the early detection of disease, prognosis, and therapy monitoring. In the last decade, mass spectrometry (MS) has fueled a surge in proteomic biomarker discovery efforts. However, only a few of the putative protein biomarkers have been taken through verification and validation steps [1,2,3,4,5], contributing to the notion that MS is a great protein biomarker discovery tool, but a difficult one to translate into clinical practice. Further supporting this argument is the fact that, thirty years after the development of electrospray (ESI) and matrix-assisted laser desorption/ionization time-of-flight (MALDI-TOF) MS, there are only about a dozen MS protein tests in clinical use today [6]. These MS tests are run under Clinical Laboratory Improvement Amendments (CLIA) regulations, and none are FDA-approved. The complexity of the current MS approaches is one detriment to wider clinical adoption; the other is the cost of MS workflows, which is much higher than the gold standard of protein clinical testing: enzymatic immunoassays (EIA). So, there is very little incentive for developing expensive clinical MS protein tests for biomarkers that can readily be analyzed with much cheaper EIAs. That is, unless such MS tests could deliver unique content that cannot be detected with EIAs. Human proteoforms may be that content.

The term proteoform has been in use from 2013, denoting all of the different molecular forms in which the protein product of a single gene can be found [7]. Those forms have previously been referred to as protein isoforms or variants. Proteoforms resulting from changes in the coding gene sequence can readily be analyzed at the gene level with sequencing technologies. However, proteoforms that arise as a result of post-translational modifications, such as the addition of chemical moieties and removal of amino acid residues, can only be deciphered at the protein level. Additionally, this is where mass spectrometry offers a clear advantage over any other technique, by measuring the unique mass-to-charge (*m*/*z*) signals of individual proteoforms. EIAs cannot detect proteoforms because the UV/Vis or fluorescence readout is not proteoform-specific. So, while the cost and complexity of current MS protein tests are factors impeding their use, the new content enabled by proteoforms detection could provide the incentive for their clinical adoption [8]. However, first, the clinical utility of these proteoforms needs to be demonstrated.

There are several examples of clinically significant proteoforms. Of the dozen clinical MS protein tests in use today, three target proteoforms [6]. The carbohydrate-deficient proteoform of transferrin, which is indicative of chronic alcohol abuse and congenital disorders of glycosylation, is being analyzed with an LC-MS test [9]. A similar MS method is used for the detection of structural changes in transthyretin that are associated with familial amyloidosis [10]. Hemoglobin proteoforms that give rise to thalassemias and hemogobinopathies are also tested with mass spectrometry [11]. The most clinically important human proteoform is glycated hemoglobin A1c, which is used for monitoring long-term glycemic control [12]. However, the tests for Hb1Ac are based on other technologies and not MS [13], even though the reference method for HbA1c measurement is based on mass spectrometry [14]. Moreover, home and point-of-care Hb1A devices have been FDA-cleared/CLIA-waived [15].

There are many more proteoforms with potential clinical significance that deserve a thorough investigation. However, such investigations have been slow to materialize, primarily due to the high cost and complexity of current proteomics methods. Nevertheless, there are some hybrid methods combining immunoaffinity protein isolation with MS detection that are well-suited for studying proteoforms. One such method is known as mass spectrometric immunoassay (MSIA) [16]. It utilizes a pipette tip fitted with a small microcolumn that is derivatized with antibody toward a target protein. The antibody should have wider specificity towards multiple proteoforms, hence the use of polyclonal antibodies is preferred (monoclonal antibodies targeting a preserved epitope in all proteoforms can also be used). These affinity tips are used to retrieve proteins from human samples in a confined and sample-loss-minimizing way, in preparation for MS analysis. More than 50 MS immunoassays for human protein biomarkers from human plasma, urine, and other biological fluids have been developed [17], including fully quantitative assays for B-type natriuretic peptide [18], insulin-like growth factor 1 [19], and other proteins [20,21,22,23,24,25,26]. Several new proteoforms have been discovered and catalogued using these assays [27,28,29,30,31]. Larger-scale population studies have also been executed, delineating the extent of the proteoforms distribution in human plasma of healthy individuals [32,33,34].

Only recently have efforts been made to study the clinical significance of some of these proteoforms using the mass spectrometric immunoassay approach described above. We have investigated clinical proteoforms correlations for several protein biomarkers, utilizing multiple clinical cohorts across the type 2 diabetes (T2D) continuum. Among some of the findings, a truncated proteoform of serum amyloid A was found to be lower in type 2 diabetes patients and to be correlated negatively with measures of glycemic and lipid control [35]. Cystatin C-truncated proteoforms were also found to be elevated in patients with diabetic chronic kidney disease [36], and it has been determined that these proteoforms have a strong negative correlation with the estimated Glomerular Filtration Rate (eGFR) [37].

Most interesting were the clinical correlation findings for several apolipoproteins proteoforms, which are reviewed below. Apolipoproteins function as structural components of lipoprotein particles, cofactors for enzymes, and ligands for cell-surface receptors. Recent studies have raised the prospects of apolipoprotein profiling for cardiovascular disease (CVD) [38] and a role for apolipoprotein C-III in CVD risk lowering has been suggested [39].

## 2. Apolipoprotein C-III

Apolipoprotein C-III (apoC-III) is a major protein of the triglyceride (TG)-rich lipoproteins and high-density lipoproteins (HDL), and is also present in low-density lipoproteins (LDL) [40]. ApoC-III regulates lipid metabolism in multiple ways, including the inhibition of lipoprotein lipolysis and receptor-mediated uptake of TG-rich lipoproteins, and the stimulation of very low-density lipoprotein (VLDL) production [41]. Circulating plasma apoC-III is comprised of 79 amino acids, with a single glycosylation site at Threonine 74 giving rise to four major proteoforms. The wild-type (cannonical) proteoform that does not contain a glycan chain is commonly referred to apoC-III_0a_. The other three all have a core glycan chain made of an O-linked disaccharide galactose linked to N-acetylgalactosamine (Gal-GalNAc). The proteoform containing just this glycan core is known as apoC-III_0b_, while those containing an additional one and two sialic acid residues are known as apoC-III_1_ and apoC-III_2_, respectively. The apoC-III proteoforms were initially studied with isoelectric focusing gels. MS was first applied to apoC-III studies in the late 1990s, resulting in the discovery of additional C-terminally truncated apoC-III proteoforms that were missing one or two alanine residues [42]. Recently, low-abundance apoC-III proteoforms containing fucose in the glycan chain have also been discovered with MS [43,44].

The author’s group developed an apoC-III mass spectrometric immunoassay and initially applied it to a small cohort of 96 healthy individuals, detecting all four major apoC-III proteoforms [32]. More recently, the test was advanced to a quantitative mode and applied to a cohort of 82 individuals, resulting in the detection and quantification of twelve apoC-III proteoforms [45]. The four main proteoforms accounted for 90% of the total apoC-III proteoforms; the rest were C-terminal alanine-cleaved proteoforms and fucosylated proteoforms (which were found in only 20% of the samples).

The apoC-III MS test was recently applied to several large clinical cohorts in order to examine the role of the proteoforms in lipid metabolism and cardiometabolic risk. In the first study, we examined the relationship between the proteoforms and the triglycerides (TG) concentrations utilizing a cohort of 204 adolescent Hispanic non-diabetic children. We found that the ratios of apoC-III_0a_, apoC-III_0b_, and apoC-III_1_ to apoC-III_2_ were significantly greater in overweight and obese groups compared to the healthy weight group [46]. Most importantly, apoC-III_2_ had negative relationship with fasting TG levels, compared to the positive relationship for the other apoC-III proteoforms.

In the second study, we examined the cross-sectional and longitudinal associations of the apoC-III proteoforms with plasma lipids using two cohorts of subjects with impaired glucose tolerance (*n* = 531) and type 2 diabetes (*n* = 296). At baseline, we found a strong negative correlation between the relative abundance of apoC-III_2_ and plasma TG concentrations that was not evident with the other three major proteoforms [47]. When comparing longitudinal changes in apoC-III proteoforms with changes in plasma lipids, we observed relationships similar to those found in the cross-sectional setting. Relative increases in apoC-III_2_ were favorably related to decreases in both TG and LDL. These observations follow closely the results from the first study, despite differences in the cohorts’ demographic and clinical characteristics. There was also a trend for fewer major adverse cardiovascular disease (CVD) events with higher apoC-III_2_. These associations were not detected, or were opposite of that for the other apoC-III proteoforms, suggesting a prognostic and possible mechanistic role for apoC-III_2_ in dyslipidemia and CVD risk.

The results from both studies suggest that the degree of sialylation appears to influence apoC-III function, and warrants further studies to delineate the functions and role of the proteoforms in lipid metabolism and cardiometabolic disease. This is especially important, since loss-of-function mutations in the apoC-III gene (APOC3) have been associated with decreased plasma TG, reduced coronary atherosclerosis, and a lower risk of ischemic CVD [48,49], findings that have spurred the development of new therapies to target this multifunctional apolipoprotein [50,51].

## 3. Apolipoproteins A-I

Apolipoprotein A-I (apoA-I) is a major protein component of HDL particles in plasma that has been implicated as a biomarker for cardiovascular disease [52]. Circulating plasma apoA-I is comprised of 243 amino acids, and is known to have numerous allelic variants. Furthermore, apoA-I can be oxidized at any of its three methionine residues [53,54]. Our group has developed a mass spectrometric immunoassay for apoA-I and was the first to report the existence of a C-terminally truncated proteoform missing a glutamine residue [28]. The same proteoform was also detected in a subsequent study in all subjects from a larger cohort of healthy individuals [32]. Using MS detection of tryptic apoA-I peptides, increased apoA-I methionine oxidation was observed in patients with type 1 diabetes [55]. MS studies also revealed that oxidation at methionine 148 (M148) impairs reverse cholesterol transport by apoA-I [56]. Using a multiple reaction monitoring (MRM) MS approach to detect specific apoA-I tryptic peptides, we have recently analyzed the apoA-I M148 oxidation in a small cohort of T2D patients and healthy controls; the T2D patients were further subgrouped depending on whether they had prior CVD events or not. The results revealed a significant increase in the relative ratio of the peptide containing oxidized M148 to the unmodified peptide in the HDL of participants with T2D and CVD, compared to participants with T2D but without CVD, or the control group without T2D [57]. Further MS studies with larger clinical cohorts are certainly needed to delineate the relationship between apoA-I methionines oxidation and vascular complications in CVD.

## 4. Apolipoproteins A-II

Apolipoprotein A-II (apoA-II) is the second most abundant protein of the HDL particles, where it plays a role in stabilizing the HDL assembly [58]. Although studies have shown that apoA-II is a strong predictor of risk for CVD, its role in lipid metabolism is less clear and requires further investigation [59]. ApoA-II is comprised of 77 amino acids, and it contains a single cysteine residue at position 6, as a result of which it circulates in plasma as a monomer, homodimer, or heterodimer with apolipoprotein D [60,61]. Our group has developed a mass spectrometric immunoassay for apoA-II and reported the existence of a C-terminally truncated proteoform missing a single glutamine residue, as well as cysteinylated apoA-II proteoform [28]. These proteoforms were also detected in a subsequent study with a larger cohort of healthy individuals, along with an additional N-terminal Q-cyclization proteoform [32]. Using the same MS test, we have recently analyzed the apoA-II proteoforms in a small cohort of T2D patients and healthy controls. A total of 12 apoA-II proteoforms were detected, derived by various combinations of C-terminal truncations and/or oxidation at the single methionine residue at position 26 [62]. We found that the ratios of the oxidized monomer and all oxidized proteoforms to the native apoA-II were significantly greater in the diabetic group than in the control (non-diabetic) group. These findings warrant further studies with larger clinical cohorts to better understand the role of oxidized apoA-II in diabetes.

## 5. Conclusions

The recent apolipoprotein proteoforms studies described above are the first concerted efforts to evaluate their clinical utility. Future studies of this kind are needed to expand our understanding of apolipoproteins pathophysiology and their impact on lipid metabolism and cardiometabolic risk. Further refinement of the MS tests towards simpler, lower-cost workflows can further facilitate such clinical research studies. Similar tests for other proteoforms can also be developed, greatly expanding our ability to study human proteoforms and determine their utility as potential clinical biomarkers for disease diagnosis, risk stratification, and therapy decisions.
